# Efficient Preparation of Li_2_FeSiO_4_/C with High Purity and Excellent Electrochemical Performance in Li-Ion Batteries

**DOI:** 10.3390/molecules30040808

**Published:** 2025-02-10

**Authors:** Jinhai Cui, Dezhi Chen, Mengna Xie, Yongheng Zhou, Shuai Dong, Wei Wei

**Affiliations:** 1 Henan Engineering Center of New Energy Battery Materials, School of Chemistry and Chemical Engineering, Shangqiu Normal University, Shangqiu 476000, China; 2 School of Environmental and Chemical Engineering, Nanchang Hangkong University, Nanchang 330063, China; 3 School of Petrochemical Engineering, Liaoning Petrochemical University, Fushun 113001, China; 4 School of Material and Chemical Engineering, Kaifeng University, Kaifeng 475000, China

**Keywords:** methanol solvothermal method, the new LFS precursor, atom utility efficiency, LFS/C composites, charging–discharging performance

## Abstract

One method to enhance the electrochemical performance of carbon-coated Li_2_FeSiO_4_ cathode material in lithium-ion batteries is to produce an ideal Li_2_FeSiO_4_ precursor with minimal impurities. A novel precursor for Li_2_FeSiO_4_ (Li_2_O·FeCO_3_·CH_3_OSiO_2_H) was synthesized through a methanol solvothermal reaction under stringent conditions (180 °C and 2.7 MPa), achieving a purity level of 93.2%. During synthesis, the new Li_2_FeSiO_4_ precursor exhibits unique self-purification properties and maintains a fine morphology after annealing. The resulting carbon-coated Li_2_FeSiO_4_ composites demonstrate a Brunauer–Emmett–Teller specific surface area of 102.4 m^2^/g and approximately 81% mesoporous volume, with 90% of the pore sizes measuring less than 39 nm. As a cathode material for lithium-ion batteries, this carbon-coated Li_2_FeSiO_4_ exhibits initial specific capacities of 172.3 mAh/g (charge) and 159.3 mAh/g (discharge). Remarkably, nearly 50% of the theoretical specific capacity remains after 1300 cycles at a rate of 0.1 C. The excellent electrochemical performance of the carbon-coated Li_2_FeSiO_4_ materials is demonstrated by their high lithium-ion diffusivity (D_Li+_) value of 1.26 × 10^−11^ cm^2^/s. Additionally, the enormous capacities-controlled diffusion contribution, which accounts for 70% of the total diffusion at a rate of 1C, is noteworthy. This performance can be attributed to the high purity of the carbon-free Li_2_FeSiO_4_ composite, which contains 91% Li_2_FeSiO_4_, as well as its favorable morphology.

## 1. Introduction

Due to global resource scarcity and increasing concern about the greenhouse effect, lithium-ion battery (LIB) has been widely used in the migration away from fossil fuels because of their high energy density, thermal stability, long cycle life, and lower self-discharge rate [1,2,3]. Polyanionic compounds (XO_4_)^n−^(X = Si, P, Mo, Ti, etc.), especially the commercially used LiFePO_4_, have shown advantages in safety, cost, and specific capacity when used as cathode materials [4,5,6]. As poly-oxyanion compounds, orthosilicates of carbon-coating Li_2_FeSiO_4_ (LFS/C) had been the most promising cathode material for the next generation of LIBs. Not only does it display a theoretical capacity as high as 333 mAh g^−1^ when both Li ions are extracted from the host matrix, but it also has the other advantages mentioned above [7,8,9]. However, the practical performance of LFS cathode materials is still plagued by their poor electronic conductivity and low lithium-ion diffusion coefficient (10^−14^ S cm^−1^) [10]. Reductions in the impedance of this material, including by strategies such as shrinking the particles to the nanoscale [11], coating with carbon [12], and doping with hetero-atoms [13], can increase the conductivity and lithium-ion diffusion coefficient and satisfy the need for lower charge–discharge losses in LIBs [14].

Irrespective of the mechanism, improvements in electrochemical performance are still driven by finding efficient synthesis methods for LFS composites [15]. Up to now, effective synthesis methods for LFS include combustion,1 sol–gel [16,17], hydrothermal [18], and microwave synthesis [4]. Due to a lack of separation methods, these approaches always result in impurities such as nitrate, carbonate, and carboxylate in the final LFS/C materials [19,20,21]. Although hydrothermal methods can separate LFS precursors from inorganic salts, Fe^2+^ in the LFS precursor will inevitably be oxidized to Fe^3+^ by hydrolysis [21]. A mixture of Fe_2_SiO_3_ and Li_2_O is now being used as the LFS precursor, and extra reduction must be accomplished in the subsequent calcination process with the help of a reductive carbon source such as sugar or glucose. It is not, however, easy to control the balance between degree of reduction and carbon content solely by adding reductive carbon [22].

In this paper, we propose a novel solvothermal method for preparing LFS. By using methanol as the solvent, we prevent the further oxidation of Fe^2+^ in the LFS precursor. The specific reaction conditions we employed (180 °C and a pressure of 2.7 MPa) promote CO_2_ absorption and its reaction with methanol [23,24]. As a result, the LFS precursor we obtained may contain both carboxyl and methoxy groups in addition to the traditional components such as FeO, Li_2_O, and SiO_2_. This new precursor could also provide the LFS/C cathode material with unique electrochemical performance in LIBs. To our knowledge, no reports have previously detailed this new LFS precursor or its application features. Additionally, this latest synthetic strategy offers a new approach for obtaining a range of similar polyanionic cathode materials for LIBs with optimal performance.

## 2. Results and Discussion

### 2.1. Self-Purification Effect of the New LFS Precursor

**Figure 1:**
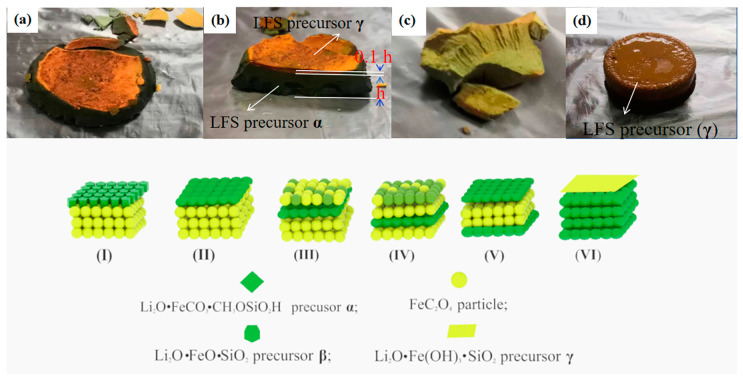
Distribution, ratio, stability, and purification effect of the LFS precursor sediments: (**a**) physical top view of LFS precursor **α** (down) and **γ** (up), (**b**) physical cross-section of LFS precursor **α** and **γ**, (**c**) partial oxidation of FS precursor **α**, and (**d**) LFS precursor **γ** obtained from hydrothermal-assisted sol-gel method; (I) sketching sediments of LFS precursor **β** (green) and FeC_2_O_4_ (yellow), (II) sketching sediments of LFS precursor **α** (green) and FeC_2_O_4_ (yellow), (III) to (V) sketching purification of LFS precursor **α** (depositing from top to bottom of sediments), (VI) sketching sediments of LFS precursor **α** (green) and **γ** (yellow).

Figure 1 illustrates the distribution, ratio, stability, and self-purification effect of sediment **α** after the solvothermal reaction. In Figure 1a, it is evident that sediment **α** can automatically separate from sediment **γ**. The dark green hue of sediment **α** at the bottom corresponds to the presence of Fe^2+^, while the orange hue of sediment **γ** in the upper layer indicates the presence of Fe^3+^. Figure 1b shows that the bulk of sediment **α** is nearly ten times larger than that of the brown sediment **γ**, suggesting that only a small amount of sediment **α** has been oxidized by the oxygen and H_2_O dissolved in methanol. In Figure 1c, sediment **α** gradually changes its hue from green to brown when exposed to air, further confirming the transformation from **α** to **γ**. Figure 1d indicates that the traditional LFS precursor obtained from the hydrothermal-assisted sol–gel process has the same brown hue as the upper sediment **γ** produced during the solvothermal reaction. Figure 1(I)–(VI) describe the self-purification effect of sediment **α** from the LFS precursor during the methanol solvothermal reaction. As shown in Figure 1(I), a partial interfacial reaction occurs between FeC_2_O_4_ and Li_2_SiO_3_, resulting in the formation of dark green sediment **β** at the top layer of the methanol solution, which is commonly known as a mixture of Li_2_O·FeO·SiO_2_. Figure 1(II)–(V) demonstrate that sediment **β** gradually transforms into sediment **α** as the interfacial reaction progresses under harsher conditions (180 °C and 2.7 MPa). Sediment **α** settles slowly at the bottom of the mixture due to its different components from sediment **β**. Ultimately, an increasing amount of sediment **α** separates from sediment **β** and sinks to the bottom of the slurry, as shown in Figure 1(V). Figure 1(VI) reveals that a small amount of sediment **β** transforms into sediment **γ** at the top layer of the slurry when oxidized by oxygen and H_2_O dissolved in methanol.

The variations among sediments **α**, **β**, and **γ** suggest that sediment **α** has a lower thermal motion rate compared to the other two sediments. This property is beneficial for achieving the self-purification effect of sediment **α**, ultimately enhancing the purity of this new precursor.

### 2.2. TGA-DTA and GC-MS Analysis for LFS Precursors

**Figure 2:**
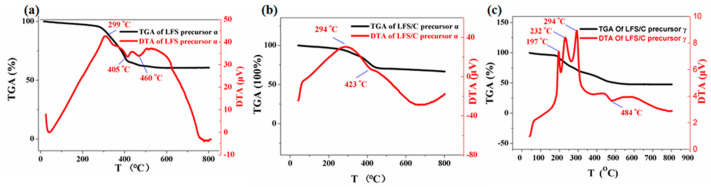
TGA-DTA analysis of (**a**) LFS precursor **α**, (**b**) LFS/C precursor **α**, and (**c**) LFS/C precursor **γ** in N_2_ atmosphere.

The thermal gravimetric analysis (TGA) and differential thermal analysis (DTA) of LFS precursors are shown in Figure 2a–c; the three LFS precursors exhibit obvious differences. Figure 2a–c show that composites LFS precursor **α**, LFS/C precursor **α**, and LFS/C precursor **γ** have DTA endothermic peaks at 299, 294, and 294 °C, respectively, accompanied by a slight TGA mass loss below 300 °C. This endothermic peak coincides with the decomposition of carbon sources such as tripropylamine and sugar that have been incorporated into the precursors during preparation [5,25].

The DTA exothermic valley at 477 °C for the LFS precursor **α** (Figure 2a) or 484 °C for the LFS/C precursor **γ** (Figure 2c) following the TGA mass loss from 300 to 500 °C indicates the beginning of the crystallization of the LFS precursor [18]. Finally, the DTA exothermic valley at 770 °C for the LFS precursor **α**, 700 °C for the LFS/C precursor **α**, and 750 °C for the LFS/C precursor **γ**, following the slow TGA mass loss from 500 to 800 °C, corresponds to crystallization of the Pmn21 phase [13].

Interestingly, Figure 1a,b also show an obvious DTA exothermic valley of 405 °C for the LFS precursor **α** and 423 °C for the LFS/C precursor **α** within the range of TGA mass loss from 300 to 500 °C. Meanwhile, there is no exothermic valley around 400 °C for the LFS/C precursor **γ** (Figure 2c). Obviously, this exothermic valley in the DTA of LFS precursor **α** and LFS/C precursor **α** around 414 °C represents a new decomposition of this precursor obtained from the methanol solvothermal method. This might indicate a structural difference between both LFS precursor **α** and LFS/C precursor **α** and the LFS/C precursor **γ** that originates from a traditional hydrothermal-assisted sol–gel process.

In any case, LFS/C precursor **γ** shows an extremely different mass loss trend and DTA profile, as seen in Figure 2c. Two extra endothermic DTA peaks at 197 and 208 °C, accompanied by a slight TGA mass loss below 300 °C, represent the loss of H_2_O and some removal of oxygen from the LFS/C precursor **γ** mixture synthesized from a mixture of Fe(NO_3_)_3_, CH_3_COOLi, and (C_2_H_5_O)_4_Si through the hydrothermal-assisted sol–gel process [22].

The purity and carbon content of the LFS precursors can be determined by analyzing the mass loss of all three precursor composites exposing either in air or nitrogen at the same annealing temperature. The results are given in Table S1 in detail. Table S1 shows that the purity of LFS/C precursor **α** can rise up to nearly 93% when their residual mass is compared after annealed in air at 700 °C (see Figure S1a,b). However, the purity of LFS/C precursor **γ** is only 66.9% based on the same calculating method (see Figure S1c). The lower purity of LFS in LFS/C precursor **γ** is coincided with the report that the traditional LFS precursor prepared through hydrothermal-assisted sol-gel always contains some carboxyl and amino impurities [22]. Table S1 also shows that the carbon content can be obtained by comparing the yield after annealing in air to that after annealing in N_2_ atmosphere at the same temperature. Based on this, the LFS/C-MA coming from LFS/C precursor **α** has a carbon content of 16.0 wt.%, and the FS/C-HA coming from LFS/C precursor **α** has a carbon content of 9.0 wt.%.

To further characterize different components in the LFS precursor **α**, GC-MS testing was used to identify the gas phase components released during annealing between 350 and 500 °C in N_2_ atmosphere. The GC-MS analysis of the annealing products of the LFS precursor **α** is shown in Figure S2. In addition to two strong peaks appearing in the chromatography (Figure S2a) at retention times of 2.532 and 2.729 min from acetone and dichloromethane in the probe rinse solution, respectively, two signal peaks at retention times of 2.215 and 2.260 min are clearly detected. The mass spectrum shown in Figure S2b suggests the gas phase component at 2.215 min is carbon dioxide (CO_2_) due to its characteristic molecular and isotope molecular ion peaks (*m*/*z*: 4 4.0 (M^+^), 45 (M+1^+^), 46 (M+2^+^)) and fragment ion peaks (*m*/*z*: 12 (C^+^), 16 (O^+^), 28 (CO^+^)). Similarly, the mass spectrum shown in Figure S2c suggests that the gas phase component at 2.260 min can be assigned to methanol (CH_3_OH) due to its characteristic molecular and isotope molecular ion peaks (*m*/*z*: 32.0 (CH_3_OH^+^), 31 (M−1^+^), 30 (M−2^+^), 29 (M−3^+^)) and fragment ion peaks (*m*/*z*: 15 (CH_3_^+^), 14 (CH_2_^+^), 13 (CH_2_^+^)).

Combining this GC–MS analysis with that of TG–DTA, it is clear that the LFS precursor **α** has a different composition from that of LFS precursor **γ** which has been prepared via many methods, such as hydrothermal reactions, microwave heating techniques, solid-state reactions, and sol–gel routes, as mentioned above. Based on the released CO_2_ and CH_3_OH and constant weight loss of 40 wt.% during sintering from the new LFS precursor, LFS precursor **α** can be verified as a mixture of Li_2_O·FeCO_3_·CH_3_OSiO_2_H components.

It is not surprising that precursor **α** contains both carboxyl and methoxy groups in the mixture during the solvothermal reaction under harsh conditions (180 °C and 2.7 MPa). It coincides with the reports that the lithium silicate can absorb both CO_2_ and CH_3_OH under certain reaction conditions [23,24]. The new LFS precursor **α** (Li_2_O·FeCO_3_·CH_3_OSiO_2_H) will then release out CO_2_ and CH_3_OH in forming the LFS cathode materials in the sintering process.

### 2.3. Reaction Mechanism of the New LFS Precursor

**Scheme 1:**
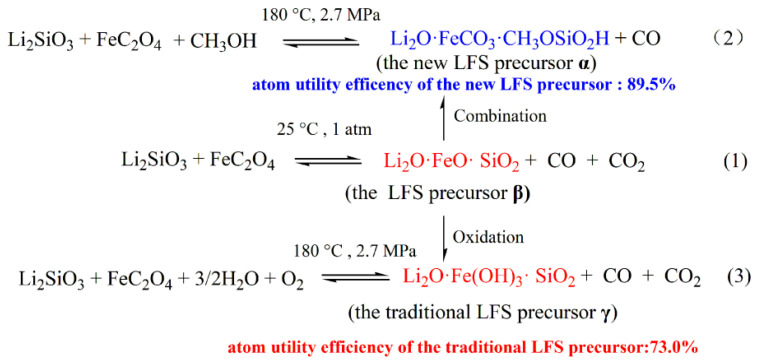
Transformation of different sediments of LFS precursor in methanol solvothermal reaction.

Scheme 1 illustrates the mechanism by which three LFS precursors, **α**, **β**, and **γ**, are formed during a methanol solvothermal reaction under varying conditions: ambient temperature and pressure (25 °C, 1 atm), elevated temperature and pressure (180 °C and 2.7 MPa), and oxidative conditions, respectively. Reaction (1) indicates that a yield of 32 wt% of the LFS precursor **β** can be achieved when the equilibrium reaction between FeC_2_O_4_ and Li_2_SiO_3_ in methanol is established at ambient temperature. In reaction (2), a new precursor, **α**, is formed with a notably high atom-utility efficiency of 89.5% when the interface reaction between FeC_2_O_4_ and Li_2_SiO_3_ is further processed in a sealed vessel under higher temperature and pressure conditions. This impressive theoretical atom-utility efficiency closely matches the practical yields—81 wt% for precursor **α** and approximately 10 wt% for precursor **γ**.

In contrast, the traditional precursor **γ** exhibits a lower theoretical atom-utility efficiency of 73.0%, as shown in reaction (3) of Scheme 1. The significant increase in atom-utility efficiency for precursor **α** suggests a self-purification effect. Factors contributing to this include the substantial bulk of precursor **α** and its reduced thermal motion rate.

Taking into account both the self-purification and the unique decomposition behavior of precursor **α**, it can be anticipated that the LFS/C-MA composite will demonstrate exceptional electrochemical performance as a cathode material in lithium-ion battery (LIB) testing.

### 2.4. Structural Analysis for LFS/C Composites

**Figure 3:**
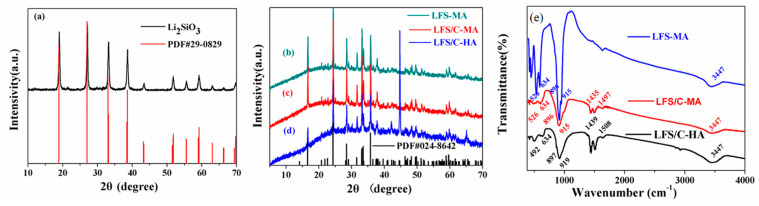
XRD patterns of (**a**) Li_2_SiO_3_ (Li_2_O:SiO_2_ = 2:1), (**b**) carbon-free LFS sample from the methanol solvothermal process labeled LFS-MA, (**c**) carbon-coated LFS sample from the methanol solvothermal process labeled LFS/C-MA, (**d**) carbon-coated LFS sample from the hydrothermal-assisted sol–gel process labeled LFS/C-HA, and (**e**) IR of LFS-MA, LFS/C-MA, and LFS/C-HA.

The XRD patterns for LFS-MA, LFS/C-MA, and LFS/C-HA, which are derived from the LFS precursor **α**, carbon-coated LFS precursor **α**, and carbon-coated LFS precursor **γ**, respectively, along with Li_2_SiO_3_, are presented in Figure 3a–d. The lattice parameters for both Li_2_SiO_3_ and LFS/C-MA, which correspond with the XRD analysis conducted by Cruz et al., are provided in Table 1 [26,27].

Li_2_SiO_3_ was synthesized via the hydrothermal method for optimal purity and uniformity. Figure 3a shows that the diffraction peaks align with ICDD standard card PDF#29-0829. The lattice constants are a = 9.392 Å, b = 5.397 Å, and c = 4.660 Å, indicating that Li_2_SiO_3_ has an orthorhombic structure with the space group Cmc21 [28].

XRD patterns of LFS-MA and LFS/C-MA prepared through the methanol solvothermal process and LFS/C-HA prepared through the hydrothermal-assisted sol–gel process are shown in Figure 3b–d, respectively. They show that all three materials have the orthogonal crystal structure due to the exact matches to the ICDD standard card PDF#97-024-8642 and can be assigned to the Pmn21 space group, consistent with recent reports [13]. Simultaneously, LFS/C-HA shows an extra strong diffraction peak near 44.8° (2θ) as shown in Figure 3d. It shows that lithium ferrosilicate (LiFeO_2_) might be present as an impurity in the LFS/C sample [11]. Considering the hydrothermal-assisted sol–gel process, it is not surprising that there is always some Fe^3+^ in the LFS/C composites due to the hydrolysis of Fe^2+^ in aqueous systems [29,30,31]. The higher purity of both LFS-MA (Figure 3b) and LFS/C-MA (Figure 3c) shows that the methanol solvothermal reaction might be a more suitable preparation method for LFS/C composites than the hydrothermal-assisted sol–gel process.

The lattice parameters and unit cell volume of Li_2_SiO_3_ and LFS/C-MA obtained from the XRD patterns are shown in Table 1.

The peaks at approximately 492 and 634 cm^−1^ in the IR spectra of LFS-MA, LFS/C-MA, and LFS/C-HA in Figure 3e can be assigned to the Li–O and Fe–O stretching modes in the LiO_4_ tetrahedra, respectively. The bands at 445 and 526 cm^−1^ belong to the (SiO_4_)^4−^ bending vibrations, and the absorption peaks at 896–897 and 915–919 cm^−1^ are from the (SiO_4_)^4−^ stretching vibrations for the three samples [32]. The IR bands at 1435–1439 and 1497–1508 cm^−1^ for LFS/C-MA and LFS/C-HA, respectively, can be attributed to a carboxylic group. In contrast, LFS-MA shows no absorption between 1437 and 1508 cm^−1^, indicating clearly that the carboxylic groups of the LFS/C composites might originate not only from the absorption of CO_2_ from air but also from the incomplete carbonization of reductive carbon sources (such as sucrose) in the LFS precursor during the calcination in inert gas [33].

**Figure 4:**
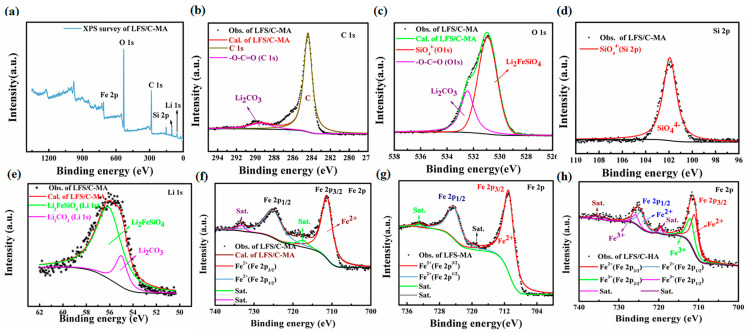
XPS spectra of LFS/C-MA: (**a**) survey and deconvoluted, (**b**) C1s, (**c**) O1s, (**d**) Si2p, (**e**) Li1s, (**f**) Fe2p; and of (**g**) LFS-MA Fe2p and (**h**) LFS/C-MA Fe2p.

X-ray photoelectron spectroscopy (XPS) was used to investigate the chemical composition and elemental valence states of the three LFS samples. The XPS survey spectra in Figure S3a, S4a, and Figure 4a confirm the presence of Li, Fe, Si, O, and C in the three LFS samples. The C1s spectrum of LFS/C-MA shown in Figure 4b contains both a major binding energy (BE) peak at 284.4 eV and a minor BE peak at 290.0 eV. The major peak originates from the carbon on the LFS particles, and the smaller peak is expected for Li_2_CO_3_ [34]. Similarly, the C1s spectra of the LFS-MA sample with a BE peak at 288.8 eV (Figure S3b) and the LFS/C-HA sample with a BE peak at 289.5 eV (Figure S4b) also show the existence of Li_2_CO_3_. This suggests that LFS has a tendency to absorb CO_2_ when exposed to air [32]. In the O1s spectrum of LFS/C-MA (Figure 4c), an intense BE peak at 530.9 eV from the oxygen in Li_2_FeSiO_4_ is clearly observed, and another weaker BE peak is present at 532.45 eV. This is from the oxygen in Li_2_CO_3_, just as the BE peak at 290.0 eV in the C1s spectrum of LFS/C-MA is from the carbon in Li_2_CO_3_, as discussed above. The O1s spectrum of LFS-MA shows comparable BE peaks at 530.5 eV (strong) and 531.5 eV (weak) (Figure S3c), and the O1s spectrum of LFS/C-HA shows comparable peaks at 530.45 eV (weak) and 531.35 eV (strong) (Figure S4c), further confirming the assignment of the oxygen peaks. Not only LFS/C-MA but also LFS-MA and LFS/C-HA have a single strong signal at 102.1 eV in the Si2p spectrum (Figure 4d, Figure S3d and S4d, respectively). All of these show that Si exists in silicate groups.

Figure 4b shows that the lithium near the surface exists in more than one state due to the double Li1s peaks of LFS/C-MA. The BE peak at 55.0 eV implies the existence of Li_2_CO_3_ at the surface of the LFS/C-MA sample and is identical to that reported by Aurbach et al. [35]. This also supports the conclusions drawn from the C1s and O1s spectra. Another strong BE peak at 56.1 eV can be definitively assigned to the Li in Li_2_FeSiO_4_. This corresponds to similar pairs of Li1s peaks at 56.1 and 54.7 eV for LFS-MA (Figure S3e) and at 56.6 and 55.1 eV for LFS/C-MA (Figure S4e).

In comparison with the complicated Fe2p spectrum of LFS/C-HA (Figure 4h), the Fe2p spectra of both LFS/C-MA (Figure 4f) and LFS-MA (Figure 4g) only have two pairs of BE peaks at 711.4 and 725.0 eV, and at 711.1 and 724.7 eV, respectively. These BE peaks correspond to Fe 2p3/2 and Fe 2p1/2 of Fe^2+^ in Li_2_FeSiO_4_ according to the literature [5,19,36]. The satellite BE peaks of LFS/C-MA (717.7 and 733.3 eV) and LFS-MA (717.4 and 733.0 eV) corroborate the attribution of Fe^2+^ in the LFS sample. Figure 4h suggests that both the Fe2p3/2 and Fe2p1/2 peaks of the LFS/C-HA sample are composed of a Fe^2+^ component on the low BE side (711.1 and 724.6 eV) and a Fe^3+^ component on the high BE side (712.2 and 725.8 eV). The single Fe^2+^ component found in both carbon-coated LFS/C-MA and carbon-free LFS-MA samples, contrasted with the coexistence of Fe^3+^ and Fe^2+^ components in the LFS/C-HA sample, might indicate that LFS samples prepared by the methanol solvothermal reaction possess a higher purity than those prepared by the hydrothermal-assisted sol–gel reaction.

**Figure 5:**
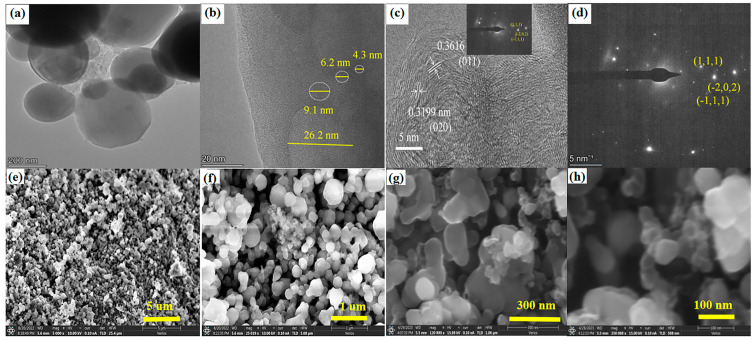
TEM images of LFS/C-MA (**a**,**b**); HRTEM image of LFS/C-MA (**c**); FFT of image of LFS/C-MA (**d**), and SEM images of LFS/C-MA (**e**–**h**).

The morphology and microstructure of the LFS/C-MA sample were investigated by TEM and SEM analysis, as shown in Figure 5a–h. The TEM images of Figure 5a,b depict the LFS/C-MA composite morphology as an accumulation of homogeneous secondary particles approximately 200 nm in diameter. These secondary particles are composed of a large number of primary particles with diameters in the range of 5–10 nm. This particle length scale is in agreement with the estimation from XRD. Figure 5c shows that the lattice interplanar distances of 0.3199 and 0.3616 nm perfectly correspond to the (020) and (011) planes of Li_2_FeSiO_4_, and the lattice fringes can be observed clearly even at a 20 nm TEM field of view (see Figure S5). Figure 5d shows the reciprocal-space lattice of LFS/C-MA at the 5 nm^−1^ scale through electron diffraction. Three diffraction spots in reciprocal space are assigned to their respective lattice planes of (111), (−202), and (−111) through the use of the Bragg formula. This assignment corresponds to an orthorhombic cell with parameters of ca. 3.8, 4.2, and 3.1 Å in direct space. The calculated axis angles of 90° indicate that the LFS/C-MA sample is orthorhombic, consistent with the space group Pmn21 determined from the XRD of LFS/C-MA in this paper.

SEM images as shown in Figure 5e–h reveal that not only are the secondary particles of LFS/C-MA the smallest in average size (approximate 100 nm) but also that they are much more loosely aggregated than layer-stacked LFS-MA (see Figure S6a–d) and agglomerated LFS/C-HA (see Figure S7a–d). The distinctive difference in aggregation of the three samples can be attributed to the different van der Waals forces between the secondary particles. In LFS/C-MA, the secondary particles combine by connecting edge to corner, edge to edge, and edge to plane in most cases, which limits the lower van der Waals forces in such accumulations. For the other two samples, the secondary particles connect plane to plane and thus agglomerate heavily due to the more powerful van der Waals forces. The much smaller particle sizes will improve the electrochemical performance of LFS/C-MA when used as the cathode material in LIBs [37,38].

**Figure 6:**
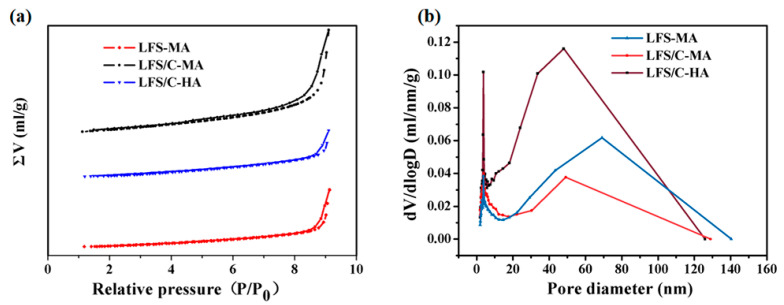
N_2_ adsorption-desorption (**a**) isotherms and (**b**) pore size distributions of LFS/C-MA, LFS/C-HA, and LFS-MA samples.

The BET adsorption–desorption isotherms and BJH pore size distributions of LFS-MA, LFS/C-MA, and LFS/C-HA are shown in Figure 6. The BJH pore size distribution at different scales is shown in Table 2. Figure 6a shows that LFS/C-MA has the largest specific surface area (102.4 m^2^ g^−1^), higher than that of LFS/C-HA (22.3 m^2^ g^−1^), while the LFS-MA sample has the lowest BET area of 18.1 m^2^ g^−1^ due to its dense stacking without a carbon coating. This difference in BET area between LFS/C-MA and LFS/C-HA shows that the methanol solvothermal method might be a better choice than other methods in the preparation of LFS/C samples.

As shown in Figure 6b and Table 2, although LFS/C-MA has the lowest mesoporous fraction of 55.8% in contrast to LFS/C-HA with the highest mesoporous fraction of 81.2% and LFS-MA with an intermediate fraction of 73.9%, the 90%-content pore diameter D90 is the smallest at 39 nm compared to LFS-MA (86 nm) and LFS/C-HA (63 nm).

The negligible effect on LFS cathode materials, which possess strong chemical and thermal stability due to robust Si–O bonding [1,2,3,31,33], indicates that optimizing their morphological structure could create an advantage in improving their electrochemical properties. Notably, the LFS/C-MA composites demonstrate a smaller particle size, a higher BET-specific surface area, and smaller pore diameters compared to the LFS-MA and LFS/C-HA composites.

### 2.5. Electrochemical Performances

**Figure 7:**
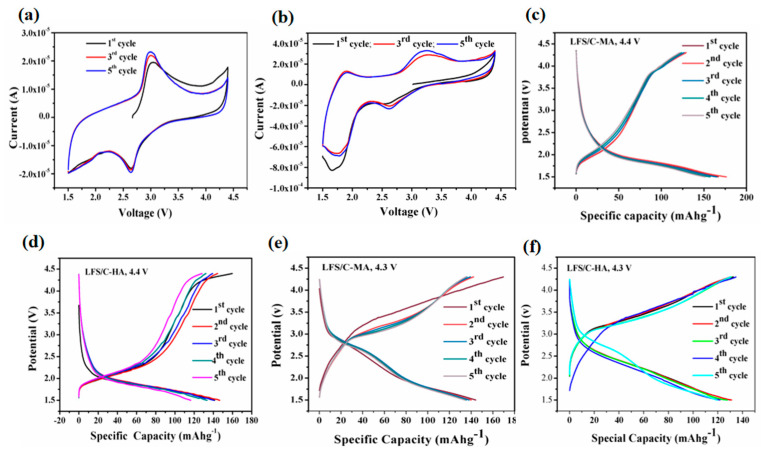
Cyclic voltammetry profiles of (**a**) LFS/C-MA and (**b**) LFS/C-HA over 1.5–4.4 V vs. Li/Li^+^ with a scanning rate of 0.2 mV s^−1^ at 20 °C, and charge–discharge profiles of (**c**) LFS/C-MA over 1.5–4.4 V, (**d**) LFS/C-HA over 1.5–4.4 V, (**e**) LFS/C-MA over 1.5–4.3 V, and (**f**) LFS/C-HA over 1.5–4.3 V at a rate of 0.1 C in half-cell LIBs.

Due to their high resistance to electronic conductivity and significantly lower diffusion coefficients in lithium-ion migration [1,2,3], only the carbon-coated materials LFS/C-MA and LFS/C-HA were assembled into half cells to demonstrate their electrochemical differences. The results are presented in Figure 7.

The initial cathodic scan began at an open circuit voltage to avoid the redox reactions of impurities activated by the lowest discharging voltage of 1.5 V. In contrast, the redox activity and structural stability of the LFS/C-MA and LFS/C-HA cathodes were examined through cyclic voltammetry. As illustrated in Figure 7a,b, the LFS/C-MA composite displays a distinct redox couple at 2.99 V and 2.63 V vs. Li/Li^+^, corresponding to the Fe^2+^/Fe^3+^ redox reaction during the charge–discharge cycle [39]. Figure 7b shows that the LFS/C-HA exhibits similar charge–discharge behavior around 3.20 V and 2.63 V vs. Li/Li^+^, but with an additional redox couple observed at approximately 1.80 V and 1.68 V vs. Li/Li^+^. This suggests that LFS/C-HA may undergo an additional charge–discharge reaction in the half-cell. Notably, a significant amount of the impurity LiFeO_2_ was detected through XRD and XPS spectra (see Figure 3d and Figure S4), making the new electrode potential of Fe^3+^/Fe^2+^—originating from the redox of LiFeO_2_—reasonable. This finding also supports the report that certain impurities in the LFS/C-HA composite can lead to side reactions within the LFS/C cathode materials [10]. Consequently, the actual specific capacity of the two LFS/C composites ultimately arises from the contributions of two distinct Fe^3+^/Fe^2+^ redox pairs. At the same time, this research did not identify another charge–discharge reaction from Fe^4+^/Fe^3+^ in the half-cell [7].

The electrochemical performance of LFS/C-MA and LFS/C-HA was evaluated and is displayed in Figure 7c–f. The first cycle charge–discharge curve in Figure 7c indicates that LFS/C-MA possesses a maximum charging specific capacity of 172.3 mAh g^−1^ and an unmatched discharging specific capacity of 159.3 mAh g^−1^ with 92% Coulombic efficiency at a rate of 0.1C. The lower Coulombic efficiency might mean that Li^+^ and Fe^2+^ have rearranged to form a Li_2_FeSiO_4_ lattice or that a side reaction of impurities occurred in the first charge–discharge cycle [40].

The subsequent Coulombic efficiencies of LFS/C-MA varied from 96.5% for the 2nd cycle to 99.9% for the 3rd cycle to 101.4% for the 4th cycle and decreased slightly to 100.2% for the 5th cycle, indicating good tolerance to the high charging voltage of 4.4 V. In contrast, the LFS/C-HA composite cathode cannot provide Coulombic efficiencies this high at the same charging voltage, as can be seen by the diminishing specific capacity in Figure 7d. Therefore, the charging voltage of the two LFS/C composites was adjusted to 4.3 V, and their profiles are shown in Figure 7e,f. Although the specific capacity for both LFS/C-MA and LFS/C-HA was reduced at a charging voltage of 4.3 V, the Coulombic efficiency was consistently between 99 and 100%. This indicates an almost complete extraction–insertion reaction of a single lithium ion for an LFS composite at a suitable charging voltage of 4.3 V.

**Figure 8:**
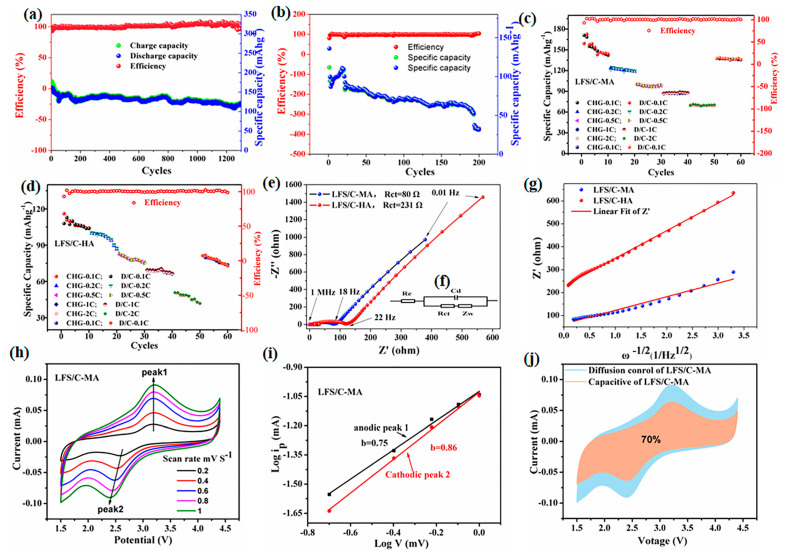
Long-term charge–discharge performance and dynamic characteristic of two LFS/C cathode materials: Efficiency and specific capacity of (**a**) LFS/C-MA, and (**b**) LFS/C-HA at a current density of 16.5 mA g^−1^ over 1.5–4.3 V at 20 °C; Cycle performance of (**c**) LFS/C-MA, and (**d**) LFS/C-HA between 1.5 and 4.3 V at different discharge rates at 20 °C; and (**e**) EIS spectra, (**f**) (inset) equivalent circuit and (**g**) profiles of the real part of impedance (Z’) vs ω^−1/2^ of both LFS/C-MA and LFS/C-HA half cells; (**h**) LCV curves of LFS/C-MA from a scan rate: 0.1 to 1 mV s^−1^, (**i**) Relationship between log (i_p_) versus log V for LFS/C-MA, and (**j**) Capacitive-controlled contributions for LFS/C-MA from 0.1 to 1 mV/s.

Figure 8a,b show that the charging–discharging lifetime of LFS/C-MA at a rate of 0.1C is greater than that of LFS/C-HA. From Figure 8a, LFS/C-MA maintains 131.2 mAh g^−1^ and 130.0 mAh g^−1^ charging and discharging specific capacities, respectively, after 200 cycles. The retained capacity is up to 80% of its theoretical specific capacity (166 mAh g^−1^). Moreover, 80.6 mAh g^−1^ and 79.4 mAh g^−1^ of the charging and discharging specific capacity is retained even after 1300 cycles. For LFS/C-HA, however, Figure 8b shows that only 31.8 mAh g^−1^ and 32.0 mAh g^−1^ of the charging and discharging specific capacity are retained even at a shorter cycling life of 200 cycles.

The exceptional long-term cycling performance of the LFS/C-MA cathode materials can be attributed to the high purity of their active components. This increased purity ensures stable electrochemical redox reactions without interference from side reactions, such as LiFeO_2_, as shown in Figure 7b. Additionally, the smaller crystallization size illustrated in Figure 5 and the higher porosity demonstrated in Figure 6 of the LFS/C-MA cathode material also significantly enhance its impressive long-term cycling performance.

The rate capabilities of LFS/C-MA and LFS/C-HA across various current densities are recorded in Figure 8c,d. They show that LFS/C-MA and LFS/C-HA lost 43.1 and 25.2%, respectively, of their theoretical specific capacity (166 mAh g^−1^) after the 50th cycle when the charge–discharge rate was increased from 0.1 (0.1 C) to 2 A g^−1^ (2C). In contrast to 71% of specific capacity of that of the tenth cycle for LFS/C-HA cathode material maintained at the 60th cycle, LFS/C-MA keeps the specific capacity of the 60th cycle recovered to 94% of that of the tenth cycle with steady Coulombic efficiency around 100% when the current rate was returned to 0.1C. It means that LFS/C-MA composites might own more excellent fast charging–discharging energy storage performance in LIBs than that of LFS/C-HA, although the two LFS materials all go through the dramatically decreased specific capacity at higher rates.

EIS analysis helps to estimate how the capability of the charge transfer and ion diffusion is decided by the resistance of electrode materials. Figure 8e shows that LFS/C-MA and LFS/C-HA have half-cell R_e_ of 6.84 and 8.84 Ω between electrode and electrolyte, respectively, based on their intercepts at the Zreal axis at high frequency, and the charge transfer resistances (R_ct_) of LFS/C-MA and LFS/C-HA cathodes are 231 and 980 Ω, respectively, which are determined from the depressed semicircles in the middle frequency range. Warburg impedances (σ_w_) of the LFS/C-MA and LFS/C-HA half cells are 379 and 736 Ω s^−1^, respectively, which are represented by the low-frequency slopes at 0.01 Hz. σ_w_ corresponds to the diffusion resistance of Li^+^ in the bulk of the LFS electrode from the electrolyte/electrode interface [41]. Although Ohmic resistance (R_e_) depends mainly on the electrolyte and fabrication parameters used throughout the experiment, the lower R_ct_ and σ_w_ of LFS/C-MA compared with LFS/C-MA show that the former has more energetic electrochemical kinetics.

The Li^+^ diffusion coefficient in the two LFS electrode materials is calculated by using the following formula [42]:

(1) DLi+=R2T22A2F4n4C2σW2

where *D_Li_*_+_ is the Li^+^ diffusion coefficient, R is the gas constant, T is the absolute temperature, n is the molar number of Fe^3+^/Fe^2+^ charges transferred during charge–discharge, A is the surface area of the electrode, F is the Faraday constant, C is the molar concentration of Li+ ions in the LFS electrode material, and σ_w_ is the Warburg factor. The values of σ_w_ can be determined by calculating the slopes of the regression lines from the plots of Z’ vs. ω^−1/2^ for LFS/C-MA and LFS/C-HA (Figure 8g).

Figure 8g shows the real part of the impedances of LFS/C-MA and LFS/C-HA in the lower frequency region of ω^−1/2^. Using σ_w_ of 59.37 for LFS/C-MA and 120.51 for LFS/C-HA, *D_Li+_* of the two cathodes during the fifth charge is found to be 2.16 × 10^−11^ cm^2^ s^−1^ and 1.66 × 10^−12^ cm^2^ s^−1^, respectively, at 4.3 V. The observed increase in D_Li+_ indicates that the LFS/C-MA material, prepared using the new methanol solvothermal method, exhibits a stronger ability to overcome the resistance to lithium-ion storage and diffusion within the cathode material compared to the LFS/C-HA material, which was prepared through the hydrothermal-assisted sol–gel process. However, the D_Li+_ values for the two LFS/C cathode materials only partially reflect their dynamic characteristics, as the low-frequency portion of the impedance spectrum does not completely align with the equivalent circuit (as shown in Figure 8f included in the insert of Figure 8e) [41,42,43].

To explore the truth of lithium ion’s storage, diffusion, and contribution from the pseudo-capacitance, those of the LCV curves, relationship between log (i_p_) versus log V, and capacity-controlled diffusion behavior of LFS/C-MA cathode materials were further investigated. As shown in Figure 8h, LFS/C-MA electrode displays a similar redox couple around 3.00 V and 2.60 V vs. Li/Li^+^ as those found in the CV testing, verifying the sole Fe^2+^/Fe^3+^ redox reaction existing in LFS/C-MA material due to its high purity. The varied peak currents both in intensity and positions with different scan rates from 0.1 to 1.0 mV s^−1^ were also chosen to calculate the adjustable parameter b through plotting method based on the Formula (3) as the following [44]:

log i_p_ = b log V + log a (2)

where i_p_ is the peak current (mA); V is the scan rate (mV S^−1^); and a and b are the adjustable parameters. Especially, the adjustable parameter b is used to category the diffusion mechanism of lithium ion in electrode material into either the diffusion-controlled (b = 0.5) or capacities-controlled process (b = 1) [44]. Figure 8i shows the b-values of the two peaks are 0.75, 0.86 responding to summit currents from peak 1 to 2, respectively. It means that a mixed lithium-ion diffusion mechanism is dominated by diffusion-controlled and surface-controlled processes [44]. Figure 8j quantitatively describes their contribution rate of the capacities-controlled diffusion to the total diffusion of lithium ion [45,46]. It indicates that the capacities-controlled diffusion dominated by pseudo-capacitance win more and more proportion in the total diffusion control for LFS/C-MA material when the scan rate increased from 0.1 to 1.0 mV s^−1^, and the utmost contribution rate can be up to 70% at scan rate of 1.0 mV s^−1^.

The capacity-controlled diffusion of LFS/C-MA at higher current rates demonstrates that it has superior long-term cycling performance and maintains a higher specific capacity compared to LFS/C-HA. This advantage is attributed to LFS/C-MA having more efficient active sites, which result from its higher purity and more effective interface reactions due to a larger BET specific surface area [47].

## 3. Materials and Method

### 3.1. Synthesis of Li_2_SiO_3_ Nanoparticles

A saturated water solution of lithium hydroxide (LiOH·H_2_O, Aladdin Biochemical Technology Co., Ltd., Shanghai, China) was dropped slowly into silica gel (30 wt.%, Rhawn Chemical Reagent Co., Ltd., Shanghai, China) and stirred for one hour. The molar ratio of LiOH·H_2_O and SiO_2_ was 2:1. The white emulsion of Li_2_SiO_3_ was then transferred into a 100 mL Teflon-lined stainless steel autoclave. The hydrothermal reactor was heated to 180 °C and held for 6 h. Li_2_SiO_3_ precipitate was then collected and dried in a vacuum oven at 60 °C for 6 h. The yield was 97.3%.

### 3.2. Synthesis of the New LFS/C Precursor

FeC_2_O_4_ (99%, Rhawn Chemical Reagent Co., Ltd., Shanghai, China), Li_2_SiO_3_, and tripropylamine (1:1:0.1 molar ratio) were dispersed in methanol at ambient temperature and stirred for 12 h. After a yellowish-green suspension emerged, the mixture was transferred to a 100 mL Teflon-lined stainless steel autoclave and heated to 180 °C for 9 h. Tripropylamine served as an auxiliary agent to disperse the raw materials in the methanol solution. At the end of the reaction, the brown slurry in the upper layer of precipitate was washed out with a small amount of methanol, and the remaining LFS precursor with a deep green hue in the bottom layer was retrieved and dried in a vacuum chamber at room temperature. The atom utility efficiency of the new LFS precursor is 81%. Two in three LFS precursors obtained in this methanol solvothermal reaction are labeled LFS precursor **α** and **β**, respectively, and LFS precursor **γ** was prepared following hydrothermal-assisted sol–gel process [22] and labeled.

### 3.3. Preparation of LFS/C Composite

The obtained LFS precursor, acetylene black (Li-2060, Denka Company Ltd., Tokyo, Japan), sucrose (99%, Tianzheng Fine Chemical Reagent Factory, Tianjin, China), and dodecyl trimethyl ammonium bromide (99%, Rhawn Chemical Reagent Co., Ltd., Shanghai China) (1:0.1:0.02: 0.05 mass ratio), were mixed in acetone and ground by ball-milling for 6 h at 300 rpm. The mixture was then separated by centrifuge at 10,000 rpm for ten minutes and dried in a vacuum chamber at room temperature. The LFS/C precursors obtained from LFS precursor **α** and **γ** are labeled LFS/C precursor **α** and LFS/C precursor **γ**, respectively.

Finally, LFS precursor **α**, LFS/C precursor **α**, and LFS/C precursor **γ** were annealed in a horizontal quartz tube furnace at 873 K for 9 h in argon with a 50 sccm min^−1^ flow rate. The obtained carbon-free and carbon-coated LFS cathode materials are labeled LFS-MA, LFS/C-MA, and LFS/C-HA, respectively.

### 3.4. Material Characterizations

Agilent 8890-5977 gas chromatography-mass spectra (GC-MS) (Agilent Technologies, Inc., Santa Clara, CA, USA) was used to characterize the gas composition during the calcination of the LFS precursor. Shimadzu DTG–60/DTG–60A thermogravimetric differential thermal analysis (TG–DTA) (Shimadzu Corp., Kyoto, Japan) was used to probe the thermal stabilities and carbon contents of the LFS/C samples ranging from ambient temperature to 800 °C both in nitrogen and air at a heating rate of 5 °C min^−1^. Nicolet 6700 Fourier transform infrared spectrometer (FT-IRs) (Thermo Fisher Scientific Inc., Waltham, MA, USA) was used to confirmed the characteristic absorption spectra of the LFS/C samples in the region of 400 to 4000 cm^−1^. Philips X’pert X-ray powder diffractometer (XRD) (PANalytical B.V., Almelo, The Netherlands) with Cu-Kα radiation was used to analyze the crystalline structure of the products. Thermo Fisher Verios G4 field emission scanning electron microscopy (SEM), energy dispersive X-ray spectroscopy (EDS) (Thermo Fisher Scientific Inc.), and JEOL JEM–2100 transmission electron microscopy (TEM) (JEOL Ltd., Tokyo, Japan) were used to characterize the morphology and microstructure. Thermo Fisher Escalab 250Xi X-ray photoelectron spectroscopy (XPS) (Thermo Fisher Scientific Inc., Waltham, MA, USA) was used to confirm elements and identify the oxidation states of elements in the material. N_2_ gas sorption/desorption isotherm at 77 K, Brunauer–Emmett–Teller (BET) specific surface area, and Barret–Joyner–Halenda (BJH) pore size distribution were measured on a 3H–2000PM specific surface and pore size analyzer (Beishide Instrument Technology Co., Ltd., Beijing, China).

### 3.5. Preparation of the LFS/C Electrode and Electrochemical Test

LFS/C was mixed with acetylene black and polyvinylidene fluoride at a mass ratio of 8:1:1 and ground manually in a jade mortar with a pestle for 0.5 h. A slurry was obtained by adding a few drops of N-methyl pyrrolidinone to the powder, and an Al current collector was uniformly coated with the slurry and dried in a vacuum oven at 120 °C for 6 h. The coated aluminum foil was cut into round disks with diameters of 14 mm as the working electrodes. The typical active material loading was 1.2–3.5 mg cm^−2^. A lithium disk and Celgard 2400 polypropylene porous membrane were used as the counter electrode and separator, respectively. A commercial solution of 1 M LiPF_6_ dissolved in ethylene carbonate/dimethyl carbonate/diethyl carbonate (EC/DMC/DEC, 1:1:1 by volume) was selected as the electrolyte. Half-cells of LFS/C electrodes were assembled in a Vigor Ar-filled glovebox (Vigor Technologies Ltd., Shenzhen, China) with oxygen and water levels <2 ppm. A CT2001A battery test system (Landian Electronics Co., Ltd., Wuhan, China) assessed the performance of cells at different charge–discharge rates in the voltage range of 1.5–4.4 V. A Model CHI660E electrochemical workstation (CH Instruments, Inc., Bee Cave, TX, USA) with a three- electrode system was used for the cyclic voltammetry (CV) test at a scan rate of 0.02 mV s^−1^ and for the electrochemical impedance spectroscopy (EIS) test of anode materials over a frequency ranging from 0.01 to 10 MHz with a voltage perturbation ΔV = 1 mV. The reported specific capacities are quoted with respect to the mass of the LFS active material. Electrode materials and chemical reagents except acetylene black mentioned above were purchased from Duoduo Reagent Network (https://www.dodochem.com, Shanghai, China) with purity of no less than 99%.

## 4. Conclusions

A new LFS precursor, designated as **α**, was synthesized using a methanol solvothermal method at elevated temperature and pressure, demonstrating self-purification behavior. The presence of both carbonyl and methoxyl components in this LFS precursor was confirmed through atom-utility efficiency, thermogravimetric analysis (TGA), differential thermal analysis (DTA), and gas chromatography-mass spectrometry (GC-MS). The three LFS precursors, **α**, **β**, and **γ**, can transform into one another under different conditions during the reaction. The resulting carbon-free LFS obtained from precursor **α** exhibited a higher purity of 93.2% compared to 66.9% from precursor **γ**. The LFS/C-MA composite derived from precursor **α** had the smallest average particle size of 100 nm and a BET-specific surface area of 102.4 m^2^/g. This composite also exhibited a higher lithium-ion diffusivity (D_Li+_) value of 10^−11^ cm^2^/s, an enormous capacities-controlled diffusion contribution (70% the total diffusion) at a rate of 1C, and a longer cycling life of 1300 cycles at a rate of 0.1C, operating between 1.5 and 4.3 V in LIBs. The methanol solvothermal reaction that produces pure-phase LFS precursors and the resulting LFS/C cathode material with an optimal BET surface area is crucial for achieving its excellent electrochemical performance.

## Figures and Tables

**Table 1:** Unit cell parameters of Li_2_SiO_3_ and LFS/C-MA.

Li_2_SiO_3_			LFS/C		
Parameter	Value	Unit cell	Parameter	Value	Unit cell
		Volume, Å^3^			Volume, Å^3^
a	9.392	236.2	a	8.2433	336.8
b	5.397		b	5.0226	
c	4.66		c	8.2373	
beta	90.00		beta	98.88	

**Table 2:** BJH pore size distributions of three samples in different scale.

Samples	S A * (m^2^/g)	APV ** (mL/g)	APD *** (nm)	D10 (nm)	D90 (nm)	M/TP **** (%)
LFS/C-MA	102.4	0.4272	9.0098	3.0644	39.1121	0.8117
LFS/C-HA	22.3	0.1122	9.0349	2.9086	62.5805	0.7389
LFS-MA	18.12	0.1265	12.2151	3.4327	85.5500	0.5581

* SA: Specific Area; ** APV: Accumulated Pore Volume; APD: *** Average Pore Diameter; **** M/TP: Mesoporous/Total pores.

## Data Availability

The original contributions presented in this study are included in the article/Supplementary Material. Further inquiries can be directed to the corresponding authors.

## Supplementary Materials

The following supporting information can be downloaded at: https://www.mdpi.com/article/10.3390/molecules30040808/s1, Figure S1. (a) TGA-(DTA) of three LFS precursor: (a) LFS-M A precursor α (air), (b) TGA-(DTA of LFS-C-M A precursor α(air), and LFS-C-HA precursor γ (air). Figure S2. Gas chromatography-mass spectra of gas components of the decomposed LFS/C precursor α within 350 and 500 °C in nitrogen atmosphere*. (a) GC spectrum of decomposed LFS/C precursor; (b) MS spectrum of CO_2_ from decomposed LFS/C precursor; (c) MS spectrum of MeOH from decomposed LFS/C precursor. Figure S3. XPS spectra of LFS-MA: (a) survey and deconvoluted, (b) C1s, (c) O1s, (d) Si2p, (e) Li1s. Figure S4. XPS spectra of LFS/C-HA: (a) survey and deconvoluted, (b) C1s, (c) O1s, (d) Si2p, (e) Li1s. Figure S5. FFT of image of LFS/C-MA. Figure S6. SEM images of LFS-MA (a–d). Figure S7. SEM images of LFS/C-HA (a–d). Table S1. purity and carbon content of LFS composites.
