# Supplementary material for: Efficient Preparation of Li2FeSiO4/C with High Purity and Excellent Electrochemical Performance in Li-Ion Batteries
Source: Molecules. 2025 Feb 10;30(4):808. doi: 10.3390/molecules30040808 (PMC11858047; doi:10.3390/molecules30040808)
Supplement: Supplementary file 1 [file molecules-30-00808-s001.zip › molecules-3448910-supplementary.pdf]

Supplementary

For

Efficient Preparation of Li_2FeSiO_4/C with High Purity and Excellent Electrochemical Performance in Li-Ion Batteries

Jinhai Cui 1,*, Dezhi Chen 2, Mengna Xie 3, Yongheng Zhou 4, Shuai Dong 1 and Wei Wei 1,*
1Henan Engineering Center of New Energy Battery Materials, School of Chemistry and Chemical Engineering, Shangqiu Normal University, Shangqiu 476000, China
2School of Environmental and Chemical Engineering, Nanchang Hangkong University, Nanchang 330063, China
3School of Petrochemical Engineering, Liaoning Petrochemical University, Fushun 113001, China
4School of Material and Chemical Engineering, Kaifeng University, Kaifeng 475000, China
*Correspondence: cuijinghai99@163.com (J.C.), weiweizzuli@163.com (W.W.)

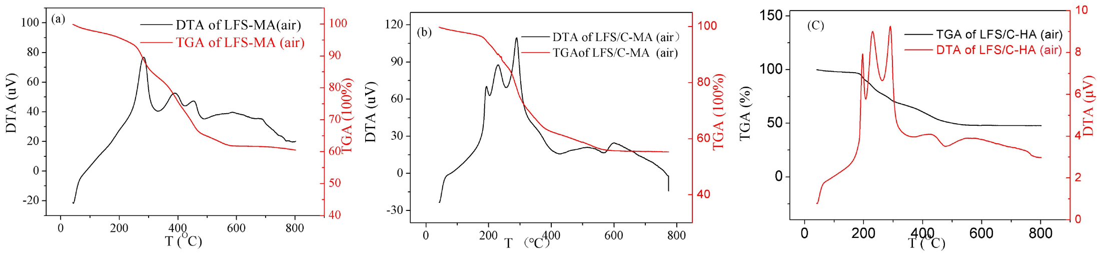

Figure S1. (a) TGA- (DTA) of three LFS precursor: (a) LFS-M A precursor **α** (air), (b) TGA-(DTA of LFS-C-M A precursor **α**(air), and LFS-C-HA precursor **γ** (air)

Table S1. purity and carbon content of LFS composites

samples / parameters	TG-DTA testing (Air)			TG-DTA testing (Nitrogen)		
	LFS-MA	LFS/C-MA	LFS/C-HA	LFS-MA	LFS/C-MA	LFS/C-HA
Pre-calcined Weight (mg)	7.535	7.071	8.525	3.273	8.012	8.991
Content of carbon source (%)	0	9.090	20	0	9.09	20
Weight of LFS precursor[a] (mg)	7.535	6.428	6.82	3.273	7.283	7.129
Theoretical weight of LFS (mg)	5.124	4.371	6.10	2.226	4.585	4.263
Post-calcined Weight (mg)	4.885	4.255	4.079	2.043	5.457	4.688
Weight of LFS[b] (mg)	4.657	4.047	3.879	-	-	-
Yield of LFS[c] (%)	58.7	63.00	59.8	-	63.0	59.8
Purity of LFS[d] (%)	91.0	93.2	66.9	91.8	-	-
Carbon content [e] (%)	0	0	0	0	16.0	9.0

[a] Components of LFS precursors in both LFS-MA and LFS/C-MA are $Li_2O·FeCO_3·OCH_3SiO_2H$,

and components of LFS precursors in the LFS/C-HA are $Li_2O{\cdot}Fe(OH)_3{\cdot}SiO_2H$. The theoretical weight of LFS is based on the reaction of the two LFS precursors during annealing, respectively, as follows:

$Li_2O{\cdot}FeCO_3{\cdot}OCH_3SiO_2H \rightarrow Li_2FeSiO_4$ (LFS) + CO_2 + CH_3OH, and $Li_2O{\cdot}Fe(OH)_3{\cdot}SiO_2H \rightarrow Li_2FeSiO_4$ (LFS) + H_2O; [b] Weight of LFS is equal to the post-calcined weight × 0.951; [c] Yield of LFS/C samples is based on the assumption that there is no chemical reaction between the LFS precursor and carbon resources; [d] Purity of LFS is based on the ratio of the weight of LFS to Theoretical weight of LFS; [e]Carbon contents of both LFS/C-MA and LFS/C-HA are based on the formula as following:

$$\text{carbon content} = \frac{\text{(Post - calcined Weight) - (Theoretical weight of LFS)}}{\text{Post - calcined Weight}} \times 100\%$$

The purity and carbon content of the LFS precursors can be determined by analyzing the mass loss of all three precursor composites exposing either in air or nitrogen at the same annealing temperature. The results are given in Table S1 in detail. Table S1 shows that the purity of LFS/C precursor **α** can rise up to nearly 93 % when their residual mass is compared after annealed in air at 700 °C (see Fig. S1(a) and (b)). However, the purity of LFS/C precursor **γ** is only 66.9 % based on the same calculating method (see Fig. S1(c)).

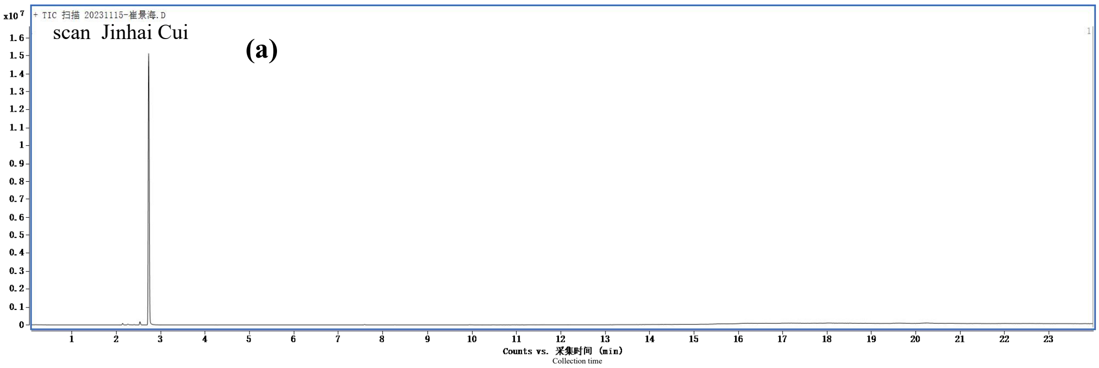

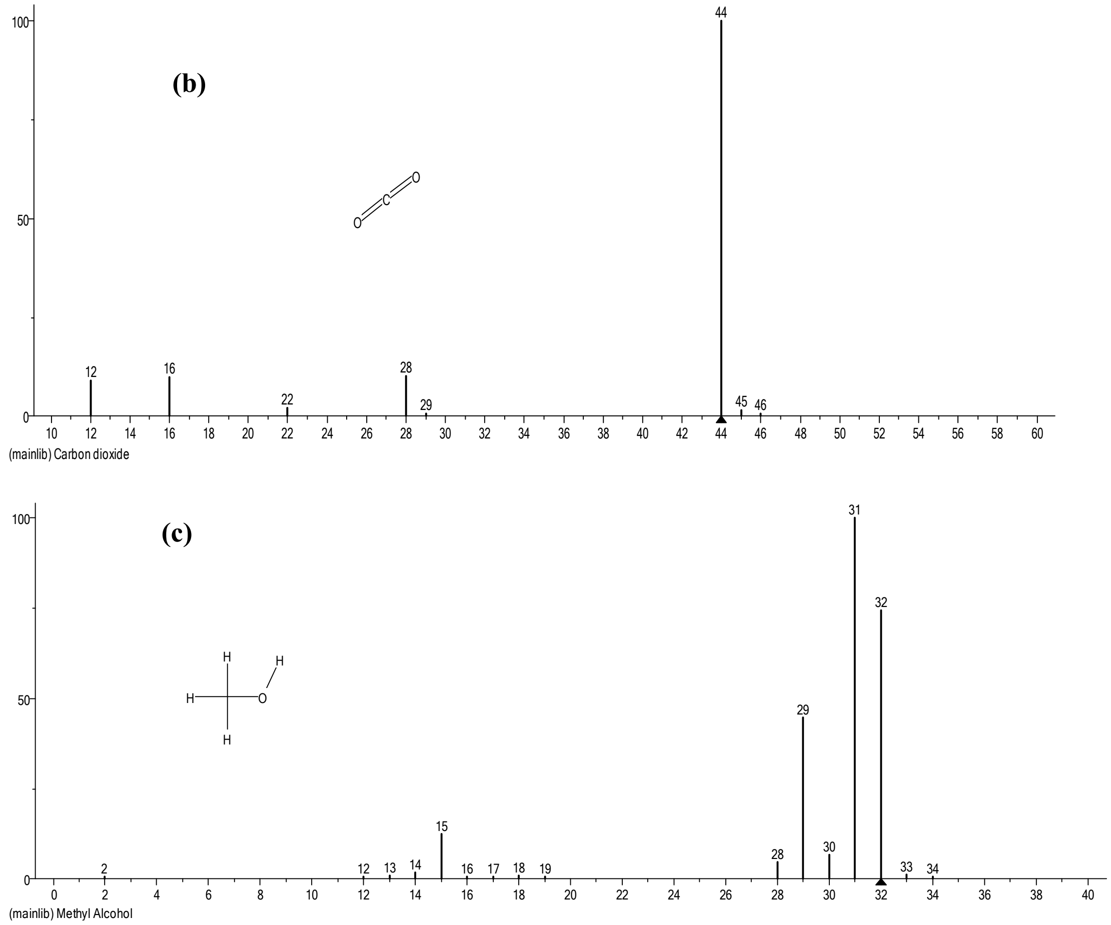

Figure S2. Gas chromatography mass spectra of gas components of the decomposed LFS/C precursor **α** within 350 and 500 °C in nitrogen atmosphere*. (a) GC spectrum of decomposed LFS/C precursor; (b) MS spectrum of CO_2 from decomposed LFS/C precursor; (c) MS spectrum of MeOH from decomposed LFS/C precursor.

*Column: HP-5 MS (30m*0.25mm*0.25um); Heating procedure: Maintain 40 °C for 5 minutes at the beginning, 40 °C /min to 280 °C; Carrier gas: 99.99% of He; flow rate: 1 mL /min; Mass spectrum ion source: EI; scanning range: 30 500; Ion source temperature: 230 °C; quadrupole pod temperature: 150 °C.

Combining this GC-MS analysis with that of TG–DTA, it is clear that the LFS precursor **α** has a different composition from that of LFS precursor **γ** which has been prepared via many methods, such as hydrothermal reactions, microwave heating techniques, solid-state reactions, and sol–gel routes as mentioned above. Based on the released CO_2 and CH_3OH and constant weight loss of 40 wt.% during sintering from the new LFS precursor, LFS precursor α can be verified as a mixture of $Li_2O \cdot FeCO_3 \cdot CH_3OSiO_2H$ components.

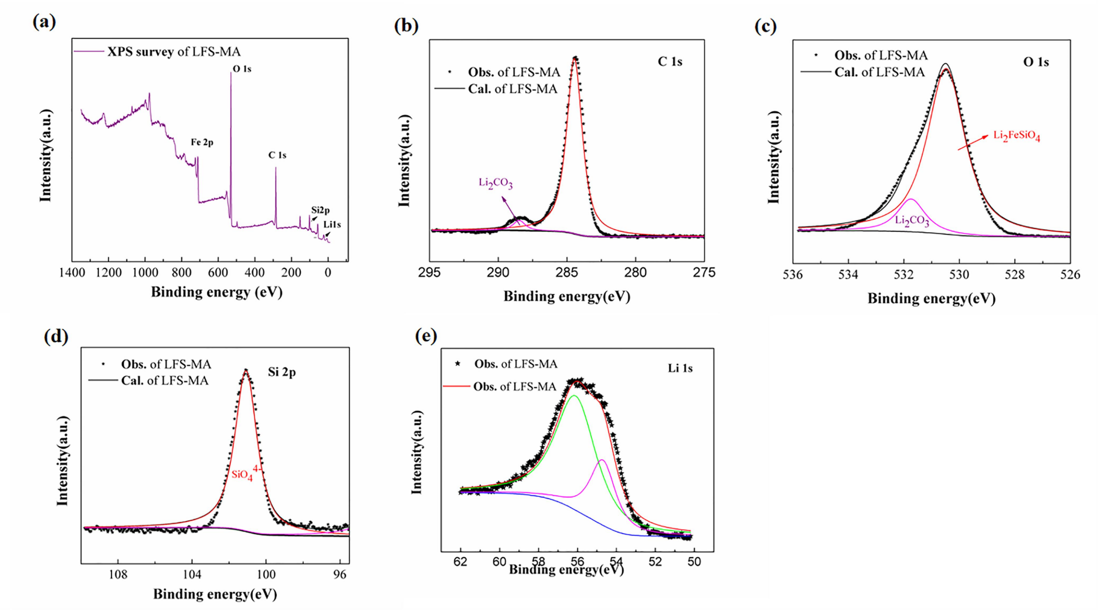

Figure S3. XPS spectra of LFS-MA: (a) survey and deconvoluted, (b) C1s, (c) O1s, (d) Si2p, (e) Li1s.

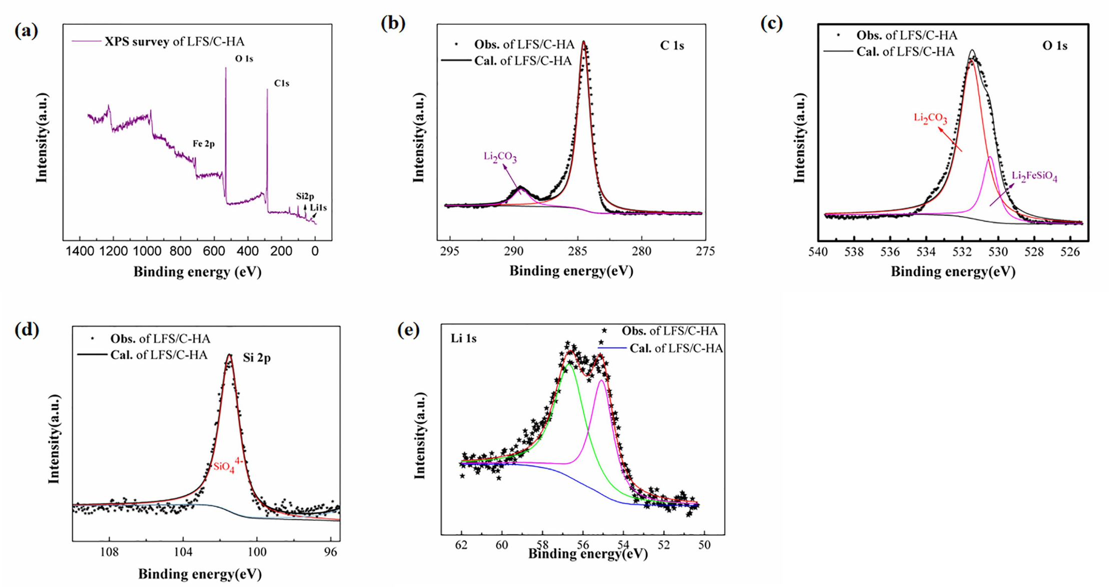

Figure S4. XPS spectra of LFS/C-HA: (a) survey and deconvoluted, (b) C1s, (c) O1s, (d) Si2p, (e) Li1s.

The XPS survey spectra in Figs. S3(a), and S4(a) confirm the presence of Li, Fe, Si, O, and C in the two LFS samples. Similarly, the C1s spectra of the LFS-MA sample with a BE peak at 288.8 eV (Fig. S3(b)) and the LFS/C-HA sample with a BE peak at 289.5 eV (Fig. S4(b)) also show the existence of Li_2CO_3. The O1s spectrum of LFS-MA shows comparable BE peaks at 530.5 eV (strong) and 531.5 eV (weak) (Fig. S3(c)), and the O1s spectrum of LFS/C-HA shows comparable peaks at 530.45 eV (weak) and 531.35 eV (strong) (Fig. S4(c)), further confirming the assignment of the oxygen peaks. Not only LFS/C-MA but also LFS-MA and LFS/C-HA have a single

strong signal at 102.1 eV in the Si2p spectrum (Figs. S3(d), and S4(d), respectively). All of these show that Si exists in silicate groups. Fig.s S3 and S4 show that the lithium near the surface also exists in more than one state due to the double Li1s BE peaks.This corresponds to similar pairs of Li1s peaks at 56.1 and 54.7 eV for LFS-MA (Fig. S3(e)) and at 56.6 and 55.1 eV for LFS/C-MA (Fig. S4(e)). It further supports the conclusion drawn from their C1s and O1s spectra.

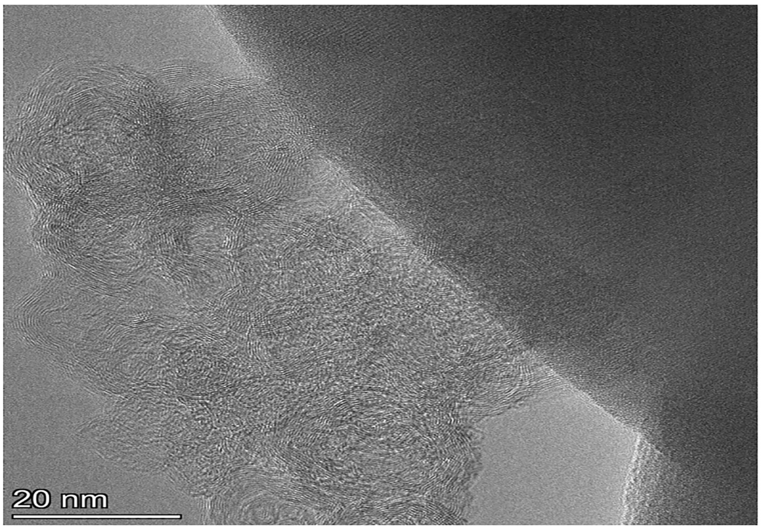

Figure S5. FFT of image of LFS/C -MA

The lattice fringes can be observed clearly even at a 20-nm TEM field of view

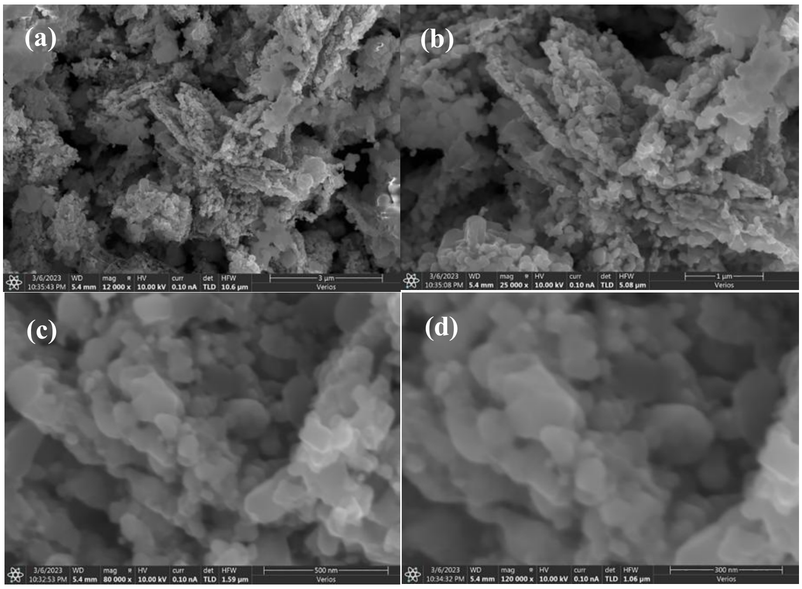

Figure S6. SEM images of LFS -MA ((a)–(d))

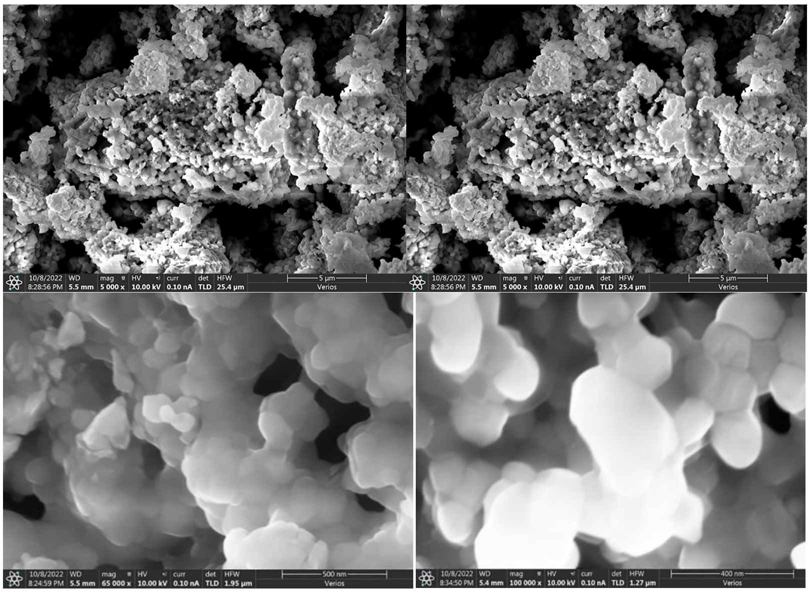

Figure S7. SEM images of LFS/C -HA ((a)–(d))

The morphology of both LFS-MA and LFS/C-HA composite show a tight assembly stacked by the layered secondary particles, and the secondary particles connect plane to plane and thus agglomerate heavily due to the more powerful van der Waals forces. Such a tight assembly will give lithium ions extra resistance to diffusing from the inner active sites.
